# Crystal structure of *fac*-tricarbon­yl(cyclo­hexyl isocyanide-κ*C*)(quinoline-2-carboxyl­ato-κ^2^ *N*,*O*)rhenium(I)

**DOI:** 10.1107/S2056989016002206

**Published:** 2016-02-17

**Authors:** Charalampos Triantis, Antonio Shegani, Christos Kiritsis, Catherine Raptopoulou, Vassilis Psycharis, Maria Pelecanou, Ioannis Pirmettis, Minas Papadopoulos

**Affiliations:** a Institute of Nuclear and Radiological Sciences and Technology, Energy and Safety, National Centre for Scientific Research ‘Demokritos’, 15310 Athens, Greece; b Institute of Nanoscience and Nanotechnology, National Centre for Scientific Research ‘Demokritos’, 15310 Athens, Greece; c Institute of Biosciences & Applications, National Centre for Scientific Research ‘Demokritos’, 15310 Athens, Greece

**Keywords:** crystal structure, rhenium(I) tricarbonyl complex, rhenium(I) cyclo­hexyl isocyanide and quinaldic acid complex, structural *trans* effect, Hirshfeld surface analysis

## Abstract

In the title compound, [Re(C_10_H_6_NO_2_)(C_7_H_11_N)(CO)_3_], the Re^I^ atom is coordinated by three carbonyl ligands in a facial arrangement and by the N, O and C atoms from a chelating quinaldate anion and a monodentate isocyanide ligand, respectively. The resultant C_4_NO coordination sphere is distorted octa­hedral. A lengthening of the axial Re—CO bond *trans* to the isocyanide ligand is indicative of the *trans* effect. Individual complexes are stacked into rods parallel to [001] through displaced π–π inter­actions. Weak C—H⋯O hydrogen-bonding inter­actions between the rods lead to the formation of layers parallel to (010). These layers are stacked along [010] by C—H⋯H—C van der Waals contacts.

## Abstract 2

The Re^I^ atom in the mol­ecule of the title compound has a distorted C_4_NO coordination sphere defined by three carbonyl ligands, one chelating quinaldate anion and one isocyanide ligand. As a result of the *trans* effect of the isocyanide derivative, one Re—CO bond is elongated.

## Chemical context   

Tri­carbonyl­rhenium(I) compounds are being explored as luminescent probes for cell imaging, photosensitizers in photocatalysis (Lyczko *et al.*, 2015 ▸; Bertrand *et al.*, 2014 ▸), and as potential radiopharmaceuticals based on the already extensive use of radioactive ^186/188^Re compounds in nuclear medicine for pain palliation and radiosynovectomy (Schneider *et al.*, 2005 ▸; Bodei *et al.*, 2008 ▸). Recent studies have also revealed the potential of cold tri­carbonyl­rhenium(I) complexes as anti­cancer agents (Leodinova & Gasser, 2014 ▸).
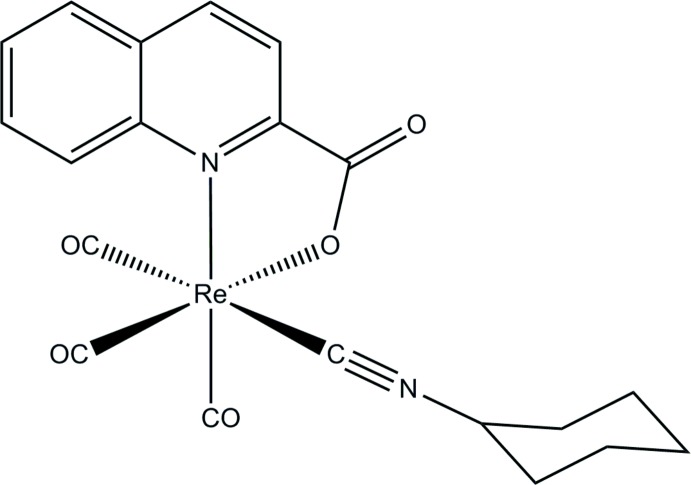


As part of our ongoing research in the field of Re/Tc coordination compounds, the crystal structure of a new ‘2 + 1’ tricarbonyl rhenium complex, *fac-*[*M*(CO)_3_(*L*)(QA-NO)], where *M* is Re,Tc, *L* is the monodentate ligand cyclo­hexyl­isocyanide, and QA-NO is deprotonated quinaldic acid, is presented. As a result of of the versatility of the ‘2 + 1’ system, *fac-*[*M*(CO)_3_(*L*)(QA-NO)] complexes can be used as model compounds in the development of targeted radiopharmaceuticals or anti­cancer agents by suitable replacement of either the bidentate or monodentate ligand. For example, the monodentate ligand may be the isocyanide derivative of a pharmacophore with affinity for a certain receptor. Alternatively, the bidentate ligand may be a more extensive conjugated system to act as a DNA inter­calator. Both quinaldate- and isocyanide-based ligands have been used as possible DNA inter­calators (Li *et al.*, 2009 ▸; Agorastos *et al.*, 2007 ▸).

## Structural commentary   

**Figure 1:**
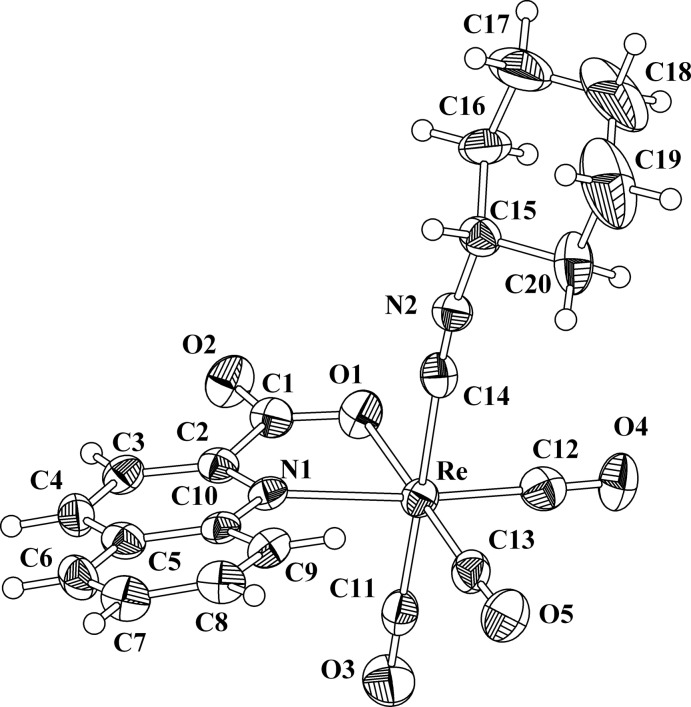
The mol­ecular structure and atom-labelling scheme of the title compound. Displacement ellipsoids are drawn at the 50% probability level.

The mol­ecular structure of the title compound, [Re(C_10_H_6_NO_2_)(C_7_H_11_N)(CO)_3_], is shown in Fig. 1 ▸. The Re^I^ atom is six-coordinated by four C, one N and one O atoms in a distorted octa­hedral coordination sphere. The carbonyl C atoms are in a facial arrangement, with distances in the range 1.903 (8)–1.960 (8) Å, resulting in a *cis* arrangement of the bi- and monodentate ligands. The longest distance involving the carbonyl ligands [1.960 (8) Å; Re—C11] corresponds to the ligand *trans* to the isocyanide cyclo­hexyl ligand, defining the axial direction of the octa­hedral complex. The Re^I^ atom almost lies in the equatorial plane (deviation, 0.006 Å) defined by the C12, C13, O1 and N1 atoms. The bite angle (N1—Re—O1) of the chelating ligand, corresponding to a five-membered ring, has a typical value of 75.2 (2)° (Lyczko *et al.*, 2015 ▸). The Re—N1 and Re—O1 bond lengths are 2.273 (5) and 2.149 (5) Å, respectively. The isocyanide carbon atom, C14, is at a distance of 2.107 (8) Å from the metal site. All these values are close to those of a complex with the same core (Agorastos *et al.*, 2007 ▸). The isocyanide group is oriented within the equatorial plane of the cyclo­hexyl ring which exhibits a chair conformation.

## Supra­molecular features   

**Figure 2:**
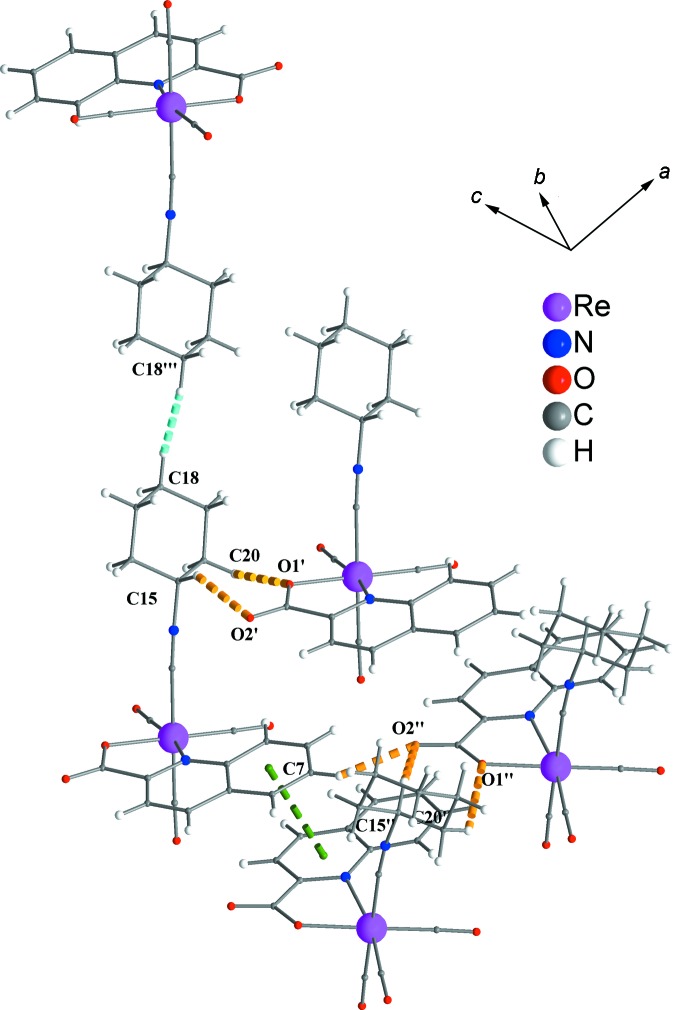
Inter­molecular inter­actions of the title complex with its neighbours. π–π inter­actions, weak C—H⋯O hydrogen bonds and short van der Waals contacts are shown with green, orange and turquoise dashed lines, respectively. [Symmetry codes: (′) *x* + 1, *y*, *z*; (′′) 1 + *x*, 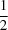 − *y*, −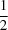 + *z*; (′′′) 4 − *x*,1 − *y*, 2 − *z*.]

**Figure 3:**
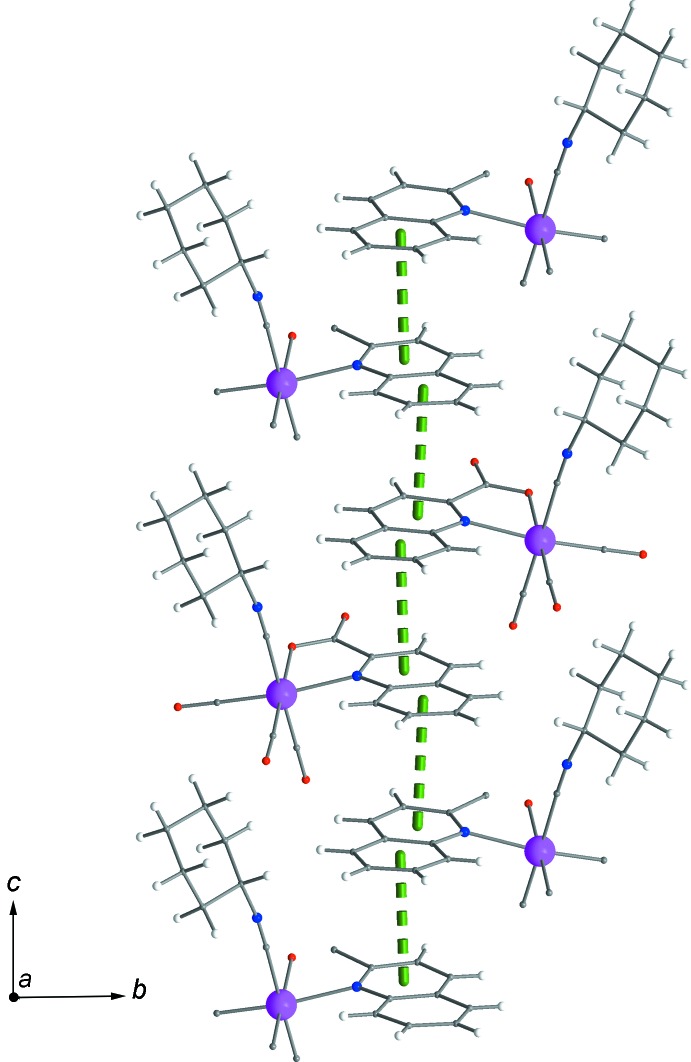
A rod of complexes extending parallel to [001] through π–π inter­actions. The colour code is as in Fig. 2 ▸.

**Figure 4:**
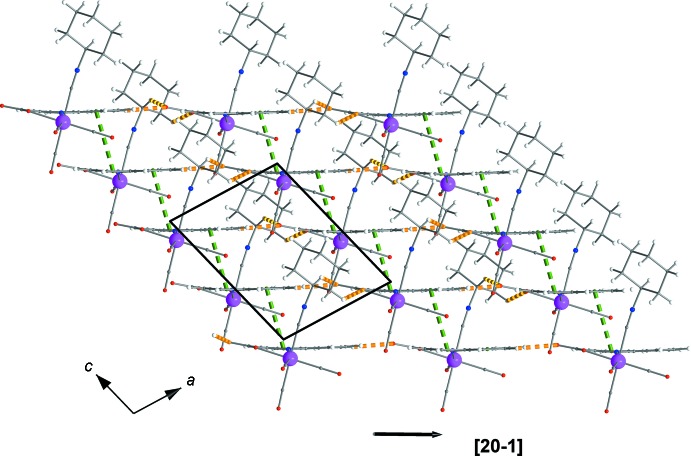
Sheet of complexes arranged parallel to (010) showing π–π and weak C—H⋯O inter­actions. The colour code is as in Fig. 2 ▸.

**Figure 5:**
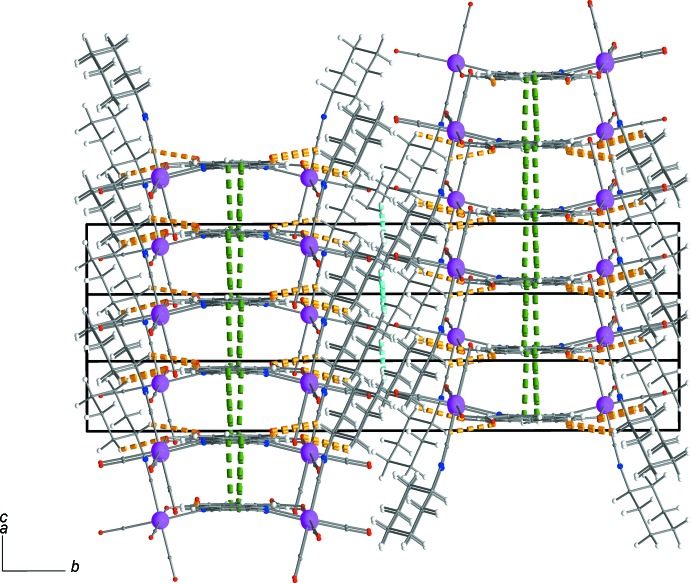
Stack of layers along [010] with C—H⋯H—C van der Waals contacts (light-blue dashed lines) developed among them, shown along the opposite [20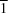] direction (see Fig. 4 ▸).

Figs. 2 ▸ and 3 ▸ show the supra­molecular inter­actions of each complex mol­ecule with its neighbours. Displaced π–π inter­actions between the phenyl and pyridine rings of quinaldate ligands of neighbouring complexes are present, with a *Cg*1⋯*Cg*2^i^ distance of 3.650 Å [*Cg*1 and *Cg*2 are the centroids of the (C5–C10) and (N1,C2,C3,C4,C5,C10) rings, respectively; symmetry code: (i): 4 − *x*, 1 − *y*, 2 − *z*]. These inter­actions help to consolidate the stacking of the mol­ecules into rods parallel to [001] (Figs. 3 ▸ and 4 ▸). Weak inter­molecular C—H⋯O hydrogen-bonding inter­actions (Table 1 ▸), including supra­molecular 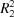
(7) loops (C20—H20*A*⋯O1 and C15—H15⋯O2) join neighbouring rods into sheets parallel to (010) (Fig. 4 ▸). An additional type of inter­actions, *viz.* short van der Waals forces of the C—H⋯H—C type (Sankolli *et al.*, 2015 ▸), is realized through C18—H18⋯H18—C18 contacts. The cyclo­hexyl end of the isocyanide ligands is hanging above and below the sheets of mol­ecules (Figs. 3 ▸ and 4 ▸), creating a perhydrogenated outer wall (Sankolli *et al.*, 2015 ▸) at both sides of the layers. Such layers are stacked along [010] (through centres of symmetry located at *b*/2) and inter­act through the aforementioned C—H⋯H—C contacts (Fig. 5 ▸).

## Hirshfeld surface analysis   

**Figure 6:**
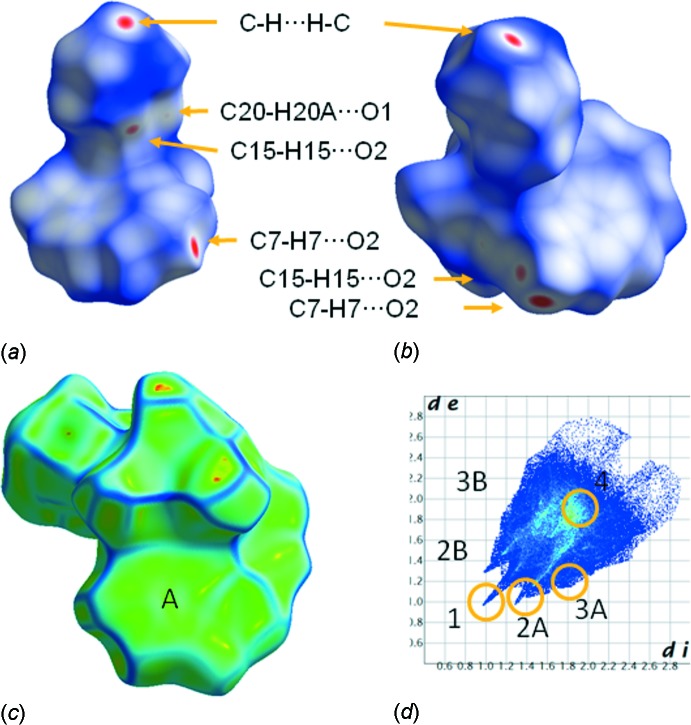
Views of Hirshfeld surfaces mapped with *d*_norm_ (*a*)/(*b*), curvedness (*c*) properties and (*d*) fingerprint plots for the title complex. *d*_e_ and *d*_i_ are the distances to the nearest atom centre exterior and inter­ior to the surface. **1**, **2**, **3** and **4** indicate H⋯H, H⋯O, C⋯H and C⋯C inter­actions, and **A** and **B** stand for acceptor and donor atoms, respectively.

The packing of the complexes in the structure was further investigated with Hirshfeld surface analysis using the *Crystal Explorer* package (Wolff *et al.*, 2012 ▸). The *d*_norm_ and curvedness (Spackman & Jayatilaka, 2009 ▸) surface mappings are presented in Fig. 6 ▸ *a*, 6*b* and 6*c*, respectively. All C—H⋯O and C—H⋯H—C contacts are recognized on the *d*_norm_ mapped surface as deep-red depression areas in Fig. 6 ▸ *a* and 6*b*, which represent two different upper views of the complex. Arrows at these figures indicate the specific type of contacts at each red point. A bottom view of the surface mapped with curvedness (Fig. 6 ▸ *c*) shows broad, relatively flat regions (indicated by letter A) characteristic of planar stacking of complexes (Spackman & Jayatilaka, 2009 ▸), corresponding to the π–π inter­actions. In the fingerprint plot (Rohl *et al.*, 2008 ▸), shown in Fig. 6 ▸ *d*, the points indicated by 1, 2A & 2B, 3A & 3B and 4 correspond to H⋯H, H⋯O, C⋯H and C⋯C inter­actions with relative contributions of 25.1, 44.2, 18.1 and 4.3%, respectively. These types of inter­actions add to 91.7% of the inter­molecular contacts of the Hirshfeld surface area. The remaining contributions (8.3%) correspond to N⋯H (2.1%), O⋯C (2.8%) and other less-important inter­actions (<1%).

## NMR investigation   

In the solution NMR spectra of the complex, both the quinaldate and iso­cyano­cyclo­hexane moieties are distinguishable. Coordination by the quinaldate is evident from the downfield shifts of all its protons ranging from 0.10 to 0.44 p.p.m. compared to free quinaldic acid under the same conditions (our data). Downfield shifts are also recorded for most of the C atoms of quinaldic acid, the most notable one (4.8 p.p.m.) being the one of the carboxyl­ate carbonyl carbon. For the iso­cyano­cyclo­hexane moiety, downfield shifts are recorded for the C atom (2.7 p.p.m.) bearing the isocyanide group and for its H atom (0.31 p.p.m.) compared to the free ligand. The most characteristic sign of coordination of the iso­cyano­cyclo­hexane moiety is the sizable upfield shift of the isocyanide C atom of 15.5 p.p.m., attributed to an increased carbene character upon coordination (Stephany *et al.*, 1974 ▸; Sagnou *et al.*, 2010 ▸, 2011 ▸). In the ^13^C NMR spectrum of the complex, one of the carbonyl ligands of the Re(CO)_3_^+^ core appears shielded (by 2.8 p.p.m. on average) compared to the other two, an observation that may also be attributed to the *trans* effect of the isocyanide ligand.

## Database survey   

A search of the Cambridge Structural Database (Groom & Allen, 2014 ▸) has revealed eight tricarbonyl complexes in facial arrangement and different *N,O*-bidentate ligands with a pyridine carboxyl­ato-2 group at the binding side of the corresponding ligand. The *N,O*-binding sites together with the two carbonyl groups *trans* to N and O atoms define an equatorial plane, and the third together with the monodentate ligand define the axial position. The Re—C bond lengths of axial carbonyl ligands (1.883–1.922 Å) *trans* to the monodentate ligand have values equal or smaller than the equatorial ones (1.892–1.945 Å) when the ligand is an aqua ligand (Schutte & Visser, 2008 ▸; Mundwiler *et al.*, 2004 ▸). The carbonyl Re—C bond lengths are inter­mediate (1.914–1.917 Å) between the values of the Re—C bonds *trans* to the equatorial O (1.886–1.916 Å) and N (1.921–1.926 Å) atoms, if the *trans* ligand is bonded to Re through an N atom (Benny *et al.*, 2009 ▸; Mundwiler *et al.*, 2004 ▸). Finally, the respective bond length, 1.947 Å, is longer than both Re—C bonds *trans* to equatorial O (1.912 Å) and N (1.914 Å) atoms if the Re atom is bonded to a P atom of a phosphine ligand (Hayes *et al.*, 2014 ▸). In the case of the isocyanide group *trans* to the axial Re—C bond (Agorastos *et al.*, 2007 ▸), the results are indistinct. In one case (XIDPUW), the axial bond length (1.756 Å) is shorter than the equatorial one (1.849 Å *trans* to O and 1.901 Å *trans* to N) whereas in the other case (XIDQAD), the corresponding length (1.914 Å) is longer than the equatorial one (1.495 Å *trans* to O and 1.885 Å *trans* to N). In the present structure, the Re—C11 bond (1.960 Å), is longer than the Re—C13 (1.903 Å, *trans* to O) and Re—C12 (1.912 Å, *trans* to N) bonds. This result is supported by the NMR analysis and is indicative of the structural *trans* effect (Coe & Glenwright, 2000 ▸).

## Synthesis and crystallization   

To a stirred solution of quinaldic acid (17.3 mg, 0.1 mmol) in 5 ml methanol, a solution of [NEt_4_]_2_[ReBr_3_(CO)_3_] (77 mg, 0.1 mmol) in 5 ml methanol was added. The mixture was heated at 333 K, and after 30 min a solution of cyclo­hexyl isocyanide (0.1 mmol) in 3 ml methanol was added. The mixture was stirred at room temperature for 2 h and the reaction progress was monitored by HPLC. The solvent was removed under reduced pressure and the solid residue was recrystallized from di­chloro­methane/hexane. The resulting solid was redissolved in a minimum volume of di­chloro­methane, layered with hexane and left to stand at room temperature. After a few days crystals suitable for X-ray analysis were isolated (yield: 44 mg, 80%). ^1^H NMR (DMSO-*d*_6_, p.p.m.): 8.93 (1H), 8.58 (1H), 8.32 (1H), 8.28 (1H), 8.18 (1H), 7.94 (1H), 4.08 (1H), 1.50 (2H), 1.40 (2H), 1.13(2H), 1.08 (2H), 0.88 (2H); ^13^C NMR (DMSO-*d*_6_, p.p.m.): 193.65, 193.12, 190.54, 172.06, 152.63, 146.23, 142.09, 138.85, 133.04, 130.47, 129.67, 129.61, 127.78, 122.78, 53.72, 30.48, 23.91, 20.70.

## Refinement   

Crystal data, data collection and structure refinement details are summarized in Table 2 ▸. C-bound H atoms were placed in idealized positions and refined using a riding model with C—H = 0.95 Å (aromatic H atoms), C—H = 0.99 Å (methyl­ene H atoms), and with *U*_iso_(H) = 1.2*U*_eq_(C).

## Supplementary Material

Crystal structure: contains datablock(s) I. DOI: 10.1107/S2056989016002206/wm5257sup1.cif

Structure factors: contains datablock(s) I. DOI: 10.1107/S2056989016002206/wm5257Isup2.hkl

CCDC reference: 1451823

Additional supporting information:  crystallographic information; 3D view; checkCIF report

## Figures and Tables

**Table 1:** Hydrogen-bond geometry (Å, °)

*D*—H⋯*A*	*D*—H	H⋯*A*	*D*⋯*A*	*D*—H⋯*A*
C20—H20*A*⋯O1^i^	0.99	2.61	3.260 (10)	123
C15—H15⋯O2^i^	0.99	2.52	3.365	142
C7—H7⋯O2^ii^	0.99	2.37	3.133	137

Symmetry codes: (i) 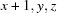; (ii) 
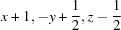
.

**Table 2:** Experimental details

Crystal data	
Chemical formula	[Re(C_10_H_6_NO_2_)(C_7_H_11_N)(CO)_3_]
*M*_r_	551.55
Crystal system, space group	Monoclinic, *P*2_1_/*c*
Temperature (K)	170
*a*, *b*, *c* (Å)	7.1529 (1), 29.5703 (5), 9.6309 (2)
β (°)	105.572 (1)
*V* (Å^3^)	1962.29 (6)
*Z*	4
Radiation type	Cu *K*α
μ (mm^−1^)	12.41
Crystal size (mm)	0.49 × 0.12 × 0.04

Data collection	
Diffractometer	Rigaku R-AXIS SPIDER *IPDS*
Absorption correction	Multi-scan (*CrystalClear*; Rigaku 2005 ▸)
*T*_min_, *T*_max_	0.374, 1.000
No. of measured, independent and observed [*I* > 2σ(*I*)] reflections	21436, 3283, 2723
*R*_int_	0.069
(sin θ/λ)_max_ (Å^−1^)	0.588

Refinement	
*R*[*F* ^2^ > 2σ(*F* ^2^)], *wR*(*F* ^2^), *S*	0.038, 0.102, 1.17
No. of reflections	3283
No. of parameters	253
H-atom treatment	H-atom parameters constrained
Δρ_max_, Δρ_min_ (e Å^−3^)	1.30, −1.43

Computer programs: *CrystalClear* (Rigaku, 2005 ▸), *SHELXS97* and *SHELXTL* (Sheldrick, 2008 ▸), *SHELXL2014* (Sheldrick, 2015 ▸), *DIAMOND* (Crystal Impact, 2012 ▸) and *PLATON* (Spek, 2009 ▸).

## supplementary crystallographic information

### Crystal data

**Table:**

[Re(C_10_H_6_NO_2_)(C_7_H_11_N)(CO)_3_]	*F*(000) = 1064
*M**_r_* = 551.55	*D*_x_ = 1.867 Mg m^−^^3^
Monoclinic, *P*2_1_/*c*	Cu *K*α radiation, λ = 1.54178 Å
*a* = 7.1529 (1) Å	Cell parameters from 17036 reflections
*b* = 29.5703 (5) Å	θ = 6.6–71.9°
*c* = 9.6309 (2) Å	µ = 12.41 mm^−^^1^
β = 105.572 (1)°	*T* = 170 K
*V* = 1962.29 (6) Å^3^	Parallelepiped, colorless
*Z* = 4	0.49 × 0.12 × 0.04 mm

### Data collection

**Table:**

Rigaku R-AXIS SPIDER IPDS diffractometer	2723 reflections with *I* > 2σ(*I*)
Radiation source: fine-focus sealed tube	*R*_int_ = 0.069
θ scans	θ_max_ = 65.0°, θ_min_ = 6.6°
Absorption correction: multi-scan (*CrystalClear*; Rigaku 2005)	*h* = −8→8
*T*_min_ = 0.374, *T*_max_ = 1.000	*k* = −33→27
21436 measured reflections	*l* = −11→11
3283 independent reflections	

### Refinement

**Table:**

Refinement on *F*^2^	0 restraints
Least-squares matrix: full	Hydrogen site location: inferred from neighbouring sites
*R*[*F*^2^ > 2σ(*F*^2^)] = 0.038	H-atom parameters constrained
*wR*(*F*^2^) = 0.102	*w* = 1/[σ^2^(*F*_o_^2^) + (0.0314*P*)^2^ + 5.6979*P*] where *P* = (*F*_o_^2^ + 2*F*_c_^2^)/3
*S* = 1.17	(Δ/σ)_max_ = 0.001
3283 reflections	Δρ_max_ = 1.30 e Å^−^^3^
253 parameters	Δρ_min_ = −1.43 e Å^−^^3^

### Special details

**Table:**

Geometry. All e.s.d.'s (except the e.s.d. in the dihedral angle between two l.s. planes) are estimated using the full covariance matrix. The cell e.s.d.'s are taken into account individually in the estimation of e.s.d.'s in distances, angles and torsion angles; correlations between e.s.d.'s in cell parameters are only used when they are defined by crystal symmetry. An approximate (isotropic) treatment of cell e.s.d.'s is used for estimating e.s.d.'s involving l.s. planes.

### Fractional atomic coordinates and isotropic or equivalent isotropic displacement parameters (Å^2^)

**Table:**

	*x*	*y*	*z*	*U*_iso_*/*U*_eq_	
Re	0.92831 (4)	0.37585 (2)	0.37372 (3)	0.03094 (14)	
N1	0.9723 (7)	0.30250 (18)	0.4300 (5)	0.0284 (12)	
O1	0.7817 (7)	0.36522 (17)	0.5387 (5)	0.0416 (12)	
O2	0.6499 (7)	0.31296 (18)	0.6478 (5)	0.0484 (13)	
C1	0.7486 (9)	0.3239 (2)	0.5673 (7)	0.0355 (16)	
C2	0.8488 (9)	0.2879 (2)	0.5021 (6)	0.0301 (14)	
C3	0.8153 (10)	0.2429 (2)	0.5234 (7)	0.0346 (15)	
H3	0.7263	0.2344	0.5762	0.042*	
C4	0.9110 (9)	0.2111 (2)	0.4680 (7)	0.0364 (16)	
H4	0.8825	0.1799	0.4757	0.044*	
C5	1.0531 (9)	0.2242 (2)	0.3988 (7)	0.0350 (16)	
C6	1.1665 (10)	0.1931 (2)	0.3467 (7)	0.0413 (17)	
H6	1.1444	0.1616	0.3549	0.050*	
C7	1.3075 (10)	0.2069 (3)	0.2848 (7)	0.0421 (18)	
H7	1.3827	0.1855	0.2496	0.051*	
C8	1.3385 (10)	0.2532 (2)	0.2742 (7)	0.0395 (17)	
H8	1.4385	0.2629	0.2332	0.047*	
C9	1.2302 (9)	0.2851 (2)	0.3207 (6)	0.0344 (15)	
H9	1.2542	0.3164	0.3112	0.041*	
C10	1.0834 (8)	0.2709 (2)	0.3826 (6)	0.0279 (14)	
C11	0.6948 (11)	0.3602 (2)	0.2213 (8)	0.0386 (16)	
O3	0.5615 (7)	0.3509 (2)	0.1301 (6)	0.0588 (16)	
C12	0.8592 (10)	0.4384 (3)	0.3514 (8)	0.0419 (17)	
O4	0.8223 (8)	0.47647 (18)	0.3391 (6)	0.0549 (14)	
C13	1.0602 (10)	0.3846 (2)	0.2285 (8)	0.0381 (17)	
O5	1.1396 (8)	0.39159 (19)	0.1404 (6)	0.0529 (13)	
C14	1.1806 (11)	0.3915 (2)	0.5378 (8)	0.0381 (16)	
N2	1.3213 (8)	0.40084 (19)	0.6200 (6)	0.0353 (13)	
C15	1.5026 (9)	0.4170 (2)	0.7131 (7)	0.0365 (16)	
H15	1.5983	0.3916	0.7321	0.044*	
C16	1.4738 (12)	0.4329 (3)	0.8551 (7)	0.057 (2)	
H16A	1.4335	0.4071	0.9061	0.068*	
H16B	1.3704	0.4561	0.8376	0.068*	
C17	1.6631 (17)	0.4527 (4)	0.9473 (10)	0.098 (4)	
H17A	1.6427	0.4645	1.0384	0.117*	
H17B	1.7629	0.4287	0.9717	0.117*	
C18	1.7339 (18)	0.4906 (4)	0.8689 (17)	0.133 (6)	
H18A	1.6382	0.5155	0.8505	0.160*	
H18B	1.8582	0.5024	0.9302	0.160*	
C19	1.7628 (14)	0.4744 (4)	0.7288 (17)	0.111 (5)	
H19A	1.8662	0.4512	0.7479	0.133*	
H19B	1.8056	0.5001	0.6788	0.133*	
C20	1.5778 (10)	0.4546 (3)	0.6321 (9)	0.055 (2)	
H20A	1.6038	0.4421	0.5437	0.066*	
H20B	1.4784	0.4786	0.6032	0.066*	

### Atomic displacement parameters (Å^2^)

**Table:**

	*U*^11^	*U*^22^	*U*^33^	*U*^12^	*U*^13^	*U*^23^
Re	0.0284 (2)	0.0315 (2)	0.0323 (2)	0.00254 (11)	0.00704 (15)	0.00250 (11)
N1	0.025 (3)	0.034 (3)	0.024 (3)	0.003 (2)	0.003 (2)	0.002 (2)
O1	0.044 (3)	0.041 (3)	0.046 (3)	0.011 (2)	0.022 (2)	0.004 (2)
O2	0.050 (3)	0.055 (3)	0.051 (3)	0.007 (3)	0.032 (3)	0.007 (3)
C1	0.031 (4)	0.037 (4)	0.036 (4)	0.007 (3)	0.006 (3)	−0.002 (3)
C2	0.023 (3)	0.035 (4)	0.029 (3)	0.002 (3)	0.001 (3)	−0.001 (3)
C3	0.038 (4)	0.040 (4)	0.029 (3)	0.001 (3)	0.015 (3)	0.003 (3)
C4	0.037 (4)	0.028 (4)	0.041 (4)	−0.001 (3)	0.007 (3)	0.002 (3)
C5	0.027 (3)	0.040 (4)	0.034 (4)	0.002 (3)	−0.001 (3)	−0.005 (3)
C6	0.044 (4)	0.029 (4)	0.048 (4)	0.007 (3)	0.007 (3)	−0.005 (3)
C7	0.033 (4)	0.049 (5)	0.046 (4)	0.007 (3)	0.014 (3)	−0.005 (4)
C8	0.031 (4)	0.049 (5)	0.040 (4)	0.005 (3)	0.012 (3)	−0.004 (3)
C9	0.027 (3)	0.044 (4)	0.032 (3)	0.002 (3)	0.008 (3)	0.006 (3)
C10	0.023 (3)	0.034 (4)	0.023 (3)	0.005 (3)	−0.001 (3)	0.001 (3)
C11	0.047 (4)	0.031 (4)	0.042 (4)	0.002 (3)	0.019 (4)	0.006 (3)
O3	0.038 (3)	0.059 (4)	0.066 (4)	−0.003 (3)	−0.010 (3)	0.001 (3)
C12	0.032 (4)	0.049 (5)	0.044 (4)	−0.006 (4)	0.008 (3)	−0.001 (4)
O4	0.063 (4)	0.036 (3)	0.067 (4)	0.010 (3)	0.021 (3)	0.013 (3)
C13	0.036 (4)	0.033 (4)	0.042 (4)	0.001 (3)	0.004 (4)	0.004 (3)
O5	0.052 (3)	0.057 (4)	0.055 (3)	−0.004 (3)	0.024 (3)	0.008 (3)
C14	0.042 (4)	0.028 (4)	0.044 (4)	0.004 (3)	0.012 (4)	0.001 (3)
N2	0.039 (3)	0.036 (3)	0.032 (3)	−0.003 (3)	0.009 (3)	−0.002 (2)
C15	0.034 (4)	0.035 (4)	0.038 (4)	0.003 (3)	0.005 (3)	−0.004 (3)
C16	0.077 (6)	0.055 (6)	0.029 (4)	0.013 (5)	−0.002 (4)	−0.005 (4)
C17	0.123 (9)	0.068 (7)	0.064 (6)	0.021 (7)	−0.042 (6)	−0.025 (5)
C18	0.093 (9)	0.073 (9)	0.172 (14)	−0.017 (7)	−0.072 (9)	−0.035 (9)
C19	0.051 (6)	0.066 (8)	0.203 (15)	−0.020 (5)	0.009 (8)	0.001 (9)
C20	0.047 (5)	0.037 (5)	0.085 (7)	−0.005 (3)	0.023 (5)	0.012 (4)

### Geometric parameters (Å, º)

**Table:**

Re—C13	1.903 (8)	C9—C10	1.404 (8)
Re—C12	1.912 (8)	C9—H9	0.9500
Re—C11	1.960 (8)	C11—O3	1.144 (8)
Re—C14	2.107 (8)	C12—O4	1.155 (8)
Re—O1	2.149 (5)	C13—O5	1.159 (8)
Re—N1	2.237 (5)	C14—N2	1.135 (8)
N1—C2	1.333 (8)	N2—C15	1.446 (8)
N1—C10	1.380 (7)	C15—C16	1.512 (9)
O1—C1	1.289 (8)	C15—C20	1.537 (9)
O2—C1	1.224 (8)	C15—H15	1.0000
C1—C2	1.510 (9)	C16—C17	1.523 (12)
C2—C3	1.376 (9)	C16—H16A	0.9900
C3—C4	1.356 (9)	C16—H16B	0.9900
C3—H3	0.9500	C17—C18	1.512 (17)
C4—C5	1.411 (9)	C17—H17A	0.9900
C4—H4	0.9500	C17—H17B	0.9900
C5—C6	1.405 (9)	C18—C19	1.496 (17)
C5—C10	1.413 (9)	C18—H18A	0.9900
C6—C7	1.365 (9)	C18—H18B	0.9900
C6—H6	0.9500	C19—C20	1.516 (13)
C7—C8	1.394 (10)	C19—H19A	0.9900
C7—H7	0.9500	C19—H19B	0.9900
C8—C9	1.371 (9)	C20—H20A	0.9900
C8—H8	0.9500	C20—H20B	0.9900
			
C13—Re—C12	87.2 (3)	C10—C9—H9	120.5
C13—Re—C11	88.4 (3)	N1—C10—C9	120.1 (6)
C12—Re—C11	90.1 (3)	N1—C10—C5	120.4 (6)
C13—Re—C14	91.6 (3)	C9—C10—C5	119.6 (6)
C12—Re—C14	90.9 (3)	O3—C11—Re	178.3 (6)
C11—Re—C14	179.1 (3)	O4—C12—Re	178.3 (7)
C13—Re—O1	179.2 (2)	O5—C13—Re	177.4 (6)
C12—Re—O1	93.5 (2)	N2—C14—Re	175.9 (6)
C11—Re—O1	91.8 (2)	C14—N2—C15	173.2 (7)
C14—Re—O1	88.1 (2)	N2—C15—C16	110.3 (6)
C13—Re—N1	104.1 (2)	N2—C15—C20	107.6 (5)
C12—Re—N1	168.7 (2)	C16—C15—C20	112.7 (6)
C11—Re—N1	89.4 (2)	N2—C15—H15	108.7
C14—Re—N1	89.7 (2)	C16—C15—H15	108.7
O1—Re—N1	75.16 (18)	C20—C15—H15	108.7
C2—N1—C10	118.3 (5)	C15—C16—C17	109.4 (8)
C2—N1—Re	111.7 (4)	C15—C16—H16A	109.8
C10—N1—Re	129.2 (4)	C17—C16—H16A	109.8
C1—O1—Re	116.8 (4)	C15—C16—H16B	109.8
O2—C1—O1	123.8 (6)	C17—C16—H16B	109.8
O2—C1—C2	119.8 (6)	H16A—C16—H16B	108.2
O1—C1—C2	116.4 (6)	C18—C17—C16	111.0 (8)
N1—C2—C3	123.8 (6)	C18—C17—H17A	109.4
N1—C2—C1	116.4 (6)	C16—C17—H17A	109.4
C3—C2—C1	119.8 (6)	C18—C17—H17B	109.4
C4—C3—C2	119.1 (6)	C16—C17—H17B	109.4
C4—C3—H3	120.4	H17A—C17—H17B	108.0
C2—C3—H3	120.4	C19—C18—C17	111.2 (10)
C3—C4—C5	119.8 (6)	C19—C18—H18A	109.4
C3—C4—H4	120.1	C17—C18—H18A	109.4
C5—C4—H4	120.1	C19—C18—H18B	109.4
C6—C5—C4	123.1 (7)	C17—C18—H18B	109.4
C6—C5—C10	118.7 (6)	H18A—C18—H18B	108.0
C4—C5—C10	118.2 (6)	C18—C19—C20	111.6 (9)
C7—C6—C5	121.7 (7)	C18—C19—H19A	109.3
C7—C6—H6	119.2	C20—C19—H19A	109.3
C5—C6—H6	119.2	C18—C19—H19B	109.3
C6—C7—C8	118.4 (7)	C20—C19—H19B	109.3
C6—C7—H7	120.8	H19A—C19—H19B	108.0
C8—C7—H7	120.8	C19—C20—C15	109.5 (8)
C9—C8—C7	122.6 (7)	C19—C20—H20A	109.8
C9—C8—H8	118.7	C15—C20—H20A	109.8
C7—C8—H8	118.7	C19—C20—H20B	109.8
C8—C9—C10	119.0 (7)	C15—C20—H20B	109.8
C8—C9—H9	120.5	H20A—C20—H20B	108.2

### Hydrogen-bond geometry (Å, º)

**Table:**

*D*—H···*A*	*D*—H	H···*A*	*D*···*A*	*D*—H···*A*
C20—H20*A*···O1^i^	0.99	2.61	3.260 (10)	123
C15—H15···O2^i^	0.99	2.52	3.365	142
C7—H7···O2^ii^	0.99	2.37	3.133	137

Symmetry codes: (i) *x*+1, *y*, *z*; (ii) *x*+1, −*y*+1/2, *z*−1/2.
